# Burnout profiles among French workers in health units for inmates: results of the EHCAU study

**DOI:** 10.1186/s12913-021-06600-3

**Published:** 2021-06-22

**Authors:** Stéphanie Boulier, Cédric Baumann, Hélène Rousseau, Pierre Horrach, Stéphanie Bourion-Bédès

**Affiliations:** 1 Centre Psychothérapique de Nancy, 54 521 Laxou, France; 2 Unit of Methodology, Data Management and Statistics, University Hospital of Nancy, 54500 Vandoeuvre-lès, Nancy, France; 3 EA4360 APEMAC (Health adjustment, measurement and assessment, interdisciplinary approaches) MICS team, University of Lorraine, 54500 Vandoeuvre-lès, Nancy, France; 4 Centre Hospitalier de Versailles, Service Universitaire de Psychiatrie de l’Enfant et de l’Adolescent, 78000 Versailles, France

**Keywords:** Professional burnout, Penitentiary environment, Workers, Health units

## Abstract

**Background:**

Health care personnel who work in penitentiary environments are at risk of burnout due to a variety of factors. Latest research have brought forward a classification system consisting of five burnout profiles on a continuum between engagement and burnout. The objective of this study was to measure the prevalence of these profiles among professionals working in French health units providing health services for inmates according to the three levels of care and to investigate their characteristics to propose appropriate management and prevention approaches.

**Methods:**

This study involved a cross-sectional analysis of data from the Evaluation of Health CAre in Units for inmates (EHCAU) study, a multicentric cohort study of professionals practising in health units for inmates in eastern France. Burnout was assessed by the Maslach Burnout Inventory (MBI) at the levels of emotional exhaustion, depersonalization and personal accomplishment. Job conditions and characteristics were measured using the Karasek Job Content Questionnaire and the Effort-Reward Imbalance Questionnaire. Data on sociodemographic characteristics and self-reported health status were also collected. Differences between MBI profiles were identified using Fisher’s exact test and the Wilcoxon test.

**Results:**

Of the 350 professionals surveyed, 150 responded (42.9%). The most frequent profiles were ineffective (36.9%) and engagement (34.8%). The burnout (7.8%), overextended (15.6%) and disengaged (5.0%) profiles made up the remaining quarter. Significant differences in the burnout profiles were observed in regard to professional occupation *(p = 0.01*), irregular eating hours (*p = 0.04*), history of complaint procedures (*p = 0.05*), anxiety (*p < 0.0001*), depression (*p < 0.0001*) and the mental component of self-reported quality of life (*p < 0.0001*).

**Conclusions:**

These results confirm that special attention should be given to professionals working in these challenging settings. The results have important implications for theory and research and for more customized approach interventions.

**Trial registration:**

ID RCB: 2018-A03029–46.

## Background

The concept of burnout in the health care system was introduced in the 1970s to describe the psychological and emotional stress experienced by clinic personnel as a result of repeated or prolonged exposure to work-related stressors [1]. Based on previous studies, burnout can be defined by emotional exhaustion (EE), feelings of cynicism (depersonalization (DP)) and a loss of meaning or purpose in work (personal accomplishment (PA)) [2–4]. Professionals and researchers have shown increased interest in studying burnout to be provided with a better understanding of what it is and how it happens [5]. Physical, psychological and occupational consequences of burnout have been reported for workers, regardless of the active population [6]. As burnout is associated with a considerable risk of both personal and/or professional consequences such as cardiovascular diseases, substance use disorders, depressive disorder, anxiety, suicide [7–10], adverse effects on quality of work, resignation and premature retirement [11, 12], practitioners need to determine ways to deal with and prevent burnout by using both individual-focused and organizational approaches [13, 14]. Furthermore, the negative effects of burnout for patient care are well known, with lower patient satisfaction, reduced professionalism with respect to medical errors, and lower viability of health care systems [1, 13]. The Maslach Burnout Inventory (MBI) was specifically designed to assess the three dimensions of the burnout syndrome. As such, it is referred to as the standard tool for research in this field [15]. The distinct burnout patterns along the burnout-engagement continuum were studied in an innovative research, reporting five different profiles based on MBI scale scores [15]. The “engagement” and “burnout” profiles are straightforward as they represent people who consistently score across the three MBI scales. The other three profiles, namely “ineffective”, “overextended” and “disengaged”, show inconsistencies across the three MBI scales. This recent approach to identifying patterns provides new opportunities for understanding both the causes and effects of burnout and may have impacts to reduce or prevent burnout by selecting the best interventions.

Between 19 and 30% of employees are affected by workplace stress and burnout in the general working population [16]. Several studies from around the world, including studies on physicians, nurses, physical and occupational therapists, primary healthcare workers and other health professionals, have reported burnout prevalence rates from 2.6 to 75% [17, 18].

Previous works assert that employment in occupations related to human services, such as health care, education and social work, is related to psychological distress [19]. Workload, job stress, role conflicts and organizational changes affect the onset of burnout. Some sociodemographic characteristics such as age, sex, marital status, educational level and years of professional experience are also known to be associated with burnout [17]. In the specific case of correctional contexts, burnout affects not only guards but also potentially the entire jail staff, including professionals in the areas of mental health and penitentiary care [20]. Psychological distress result from typical adverse conditions related to the health, safety and welfare of workers. Some stressors in the correctional workplace are constant, with the presence of demanding and hazardous working conditions, the risk of infectious diseases, irregular work shifts, reduced social and organizational support [20], violence and traumatic events with a high inmate suicide risk [21] and high job demands and low decision latitude [22].

Since 1994, the delivery of healthcare in French prisons has been managed by the Ministry of Health. This means that one neighboring hospital delivers healthcare services, with hospital departments inside every prison, under the same conditions as those experienced by free citizens [23]. Three levels of care are proposed for both somatic and psychiatric care. First-line health care in prisons (care level 1) is provided by a care unit inside the prison called the Unité de Soins en Milieu Pénitentiaire (USMP). Second-line healthcare services requiring specialized material or part-time hospitalization (care level 2) are delivered in the neighboring hospital for somatic care and the Services Médico-Psychologiques Régionaux (SMPR) units for psychiatric care. The third line of healthcare delivery includes services requiring full-time hospitalization (care level 3), and these services are delivered in an Inter-Regional Secure Hospitalized Unit (UHSI), whereas full-time psychiatric hospitalizations occur in a Specially Adapted Hospitalized Unit (UHSA) [24]. Although burnout, decreased morale, high levels of stress and staff departure are often reported among professionals in healthcare units [25–29], no previously published study has focused on the different MBI profiles among workers in these three levels of care settings in French prisons.

The first objective of our study was to investigate the prevalence of the different MBI profiles, psychological morbidity, job satisfaction and job stress among workers in health units providing services for inmates according to the three levels of care required. The second objective was to characterize the MBI profiles based on the sociodemographic characteristics of the caregivers, their professional and practice characteristics in the workplace, their job conditions, their job satisfaction and their perceived health status.

## Methods

### Participants and design

This study involved a cross-sectional analysis of data from the Evaluation of Health CAre in Units for inmates (EHCAU) study, an observational study of health care personnel working in 20 health units providing services for inmates. The specific nature of care in detention settings requires doctors, nurses, physical and occupational therapists, psychologists, hospital service agents and medical secretaries to adapt care to the needs of the population. They must be trained in specific areas, such as mental health, drug abuse, emergencies, public health and other chronic conditions. Participants were recruited from healthcare services in eastern France providing general medical care or psychiatric care in a wide range of care modalities ranging from full-time hospitalization (care level 3) to various forms of part-time (care level 2) and outpatient care (care level 1) inside the prison. The research was conducted between December 2019 and April 2020. Participation was voluntary, and informed consent was obtained from all individual participants included in the study. Consent forms were signed by each participant and kept at the main study site. The study protocol was approved by the local ethics committee, the Comité de Protection des Personnes du Sud-Ouest et Outre-Mer 4 (CPP), and ensured the confidentiality of the information collected (Comité National Informatique et Liberté 2213277v0).

When the study was launched, 350 professionals were contacted. We estimated that 60% of the professionals would respond to the survey, for a total of 210 participants. No prior sample size calculation was performed.

### Data collection

Self-report questionnaires were used to collect sociodemographic, clinical and professional data and to measure psychological distress, psychosocial job characteristics and occupational burnout.

#### Sociodemographic, clinical and professional data

Participants completed a self-administered questionnaire that included sociodemographic measures such as age, sex, marital status, number of children living at home, living arrangements, occupational status, years in profession, years caring for inmates, the level of care, work conditions and relationships with other services.

#### Health-related quality of life

The Short Form-12 questionnaire (SF-12) was used to assess the Health-related quality of life (HRQoL). It is a generic 12-item instrument based on the earlier SF-36 [30]. It covers eight domains: physical functioning, role-physical (that is, role limitations due to physical problems), bodily pain, general health, vitality, social functioning, role-emotional (that is, role limitations due to emotional problems) and mental health. The validity and reliability of the French version have been previously established [31]. A physical health component score (PCS) and a mental health component score (MCS) were calculated from all 12 items. All scores were transformed to a standardized score ranging from 0 to 100 points, with higher scores indicating better HRQoL.

#### Anxiety and depression

Anxiety and depression were assessed using the French version of the Hospital Anxiety and Depression Scale (HADS), which is a 14-item self-reporting instrument with 7 items for each subscale [32]. The French HADS questionnaire has yielded valid and reliable clinical assessments of depression and anxiety [33]. Each item is scored on a 4-point Likert scale, and the score is obtained by summing the respective 7 items for each subscale. Score range from 0 to 21. Three severity ranges based on cutoff scores are used: 0–7 (noncases), 8–10 (mild severity) and 11–21 (moderate or severe severity) [34].

#### Job conditions

Psychosocial job conditions were assessed with the Karasek Job Content Questionnaire [35]. The French 26-item version of the questionnaire measures both the psychological workload (“demands”), the level of “control” and social support. The psychometric properties of the French version have been previously reported [36]. The job demands subscale is the sum of nine items related to conflicting demands, excessive work, insufficient time to work, fast pace and hard work. The job control scale is the sum of two subscales: skill discretion (6 items) and decision authority (3 items). The work-related social support scale is the sum of two subscales: support from coworkers (4 items) and support from supervisors (4 items). For each item, the participant could choose 1 of 4 responses ranging from strongly disagree to strongly agree. The higher the score is for each scale or subscale, the higher the demand, job control and social support levels are. Job strain was defined as occurring when the professional scored low on job control and high on job demands (defined according to the median score on the respective scales). Professionals who reported low levels of social support (median split) together with job strain (high job demands and low job control) were defined as having isostrain [37].

#### Job demands and rewards

Job demands and rewards were assessed with the Effort-Reward Imbalance Questionnaire [38], for which the validity and reliability of the French version have been previously established [39]. The questionnaire includes 23 items consisting of two scales measuring the extrinsic components “effort” and “reward” and one scale measuring the intrinsic component “overcommitment”. The scale of effort includes 6 items that assess subjective feelings connected with general professional demands that refer to general and physical effort, time pressure, obstacles and responsibility. A sum of scores based on ratings of these 6 items ranged on a 5-point Likert scale from 1 (disagree) to 5 (agree and I am very distressed). A higher total score is indicative of greater efforts from professionals. The scale of rewards comprises 11 items that explore different aspects of rewards, such as financial and status-related rewards, esteem rewards and gratification of job security. Each item is scored on a 5-point Likert scale, and a sum of the ratings of these 11 items was computed. The lower the total score, the fewer rewards received by the professional. Overcommitment is measured using the sum of six items with a scale that ranges from 1 (strongly disagree) to 4 (strongly agree). The effort-reward ratio is calculated, and an imbalance between effort and reward is present when the ratio does not equal one. A ratio > 1 indicates high effort but low reward, while a ratio < 1 indicates high reward but low effort [40].

#### Burnout

Burnout was measured with the MBI scale, the validity and reliability of which have been previously established [41]. The MBI self-report questionnaire includes 22 items: 3 dimensions of EE (the feelings of being emotionally overrun and exhausted by one’s work) with 9 items, DP (the tendency to view others as objects rather than as persons with feelings) with 5 items and a lack of PA (the degree to which people perceive themselves as doing well on worthwhile tasks) with 8 items. Each response is rated on a 7-point scale (0 meaning never, 6 meaning every day). The three dimensions were measured for each participant. A higher score for EE and DP and a lower score for PA indicate a higher level of burnout. The profile characterized by favorable scores in all three dimensions is called “engagement”. Three intermediate profiles are defined as the “disengaged” profile (high DP score), the “overextended” profile (high EE score) and the “ineffective” profile (high inefficacy score) [15].

### Statistical analysis

#### Descriptive and comparative analyses

Main analysis: Continuous variables are described by the mean ± standard deviation (SD), and categorical variables are described by percentages in the full sample and according to the 3 care levels and then compared using both Fisher’s exact (categorical variables) and Mann-Whitney tests (continuous variables).

Secondary analysis: Variables are described according to the 5 MBI profiles and compared using both Fisher’s exact (categorical variables) and Mann-Whitney tests (continuous variables).

The significance level was set at 5%. Analysis was performed by SAS v9.4 (SAS Institute Inc., NC Cary, USA).

## Results

### Participant demographics and self-perceived health status scores

Of the 26 healthcare units contacted, 20 agreed to participate in the study. In total, 150 of the 350 professionals surveyed from these units returned a completed questionnaire, yielding a response rate of 42.9%. The sociodemographic and self-perceived health status of the professionals are presented in Table 1 for the entire sample and for each care level. Most professionals were female (74%), with a mean age of 42.9 years old (SD = 11.2). More than three-quarters were married or in a union (76.7%). Independent of relationship status, 78.7% of professionals reported having children, and just over half (53.4%) had at least one child living with them.

**Table 1:** Characteristics of the participants

	Full Sample		Care Level 1		Care Level 2		Care Level 3		
	*N* = 150		*N* = 78		*N* = 29		*N* = 43		
	N	%/mean (SD)	N	%/mean (SD)	N	%/mean (SD)	N	%/mean (SD)	*p value*
**Characteristic**									
**Age**	148	42.9 (11.2)	78	43.5 (11)	29	40 (12.5)	41	43.9 (10.4)	0.26
**Sex**									0.51
Male	38	25.5	21	26.9	5	17.2	12	28.6	
Female	111	74.5	57	73.1	24	82.8	30	71.4	
**Marital status**									0.10
Never married	21	14	10	12.8	8	27.6	3	7	
Married/live with a partner	115	76.7	58	74.4	20	69	37	86	
Separated/divorced/widowed	14	9.3	10	12.8	1	3.4	3	7	
**Living arrangements**									0.34
Alone	15	10.1	8	10.3	5	17.2	2	4.9	
Alone with children	13	8.8	8	10.3	4	13.8	1	2.4	
Alone with spouse	51	34.5	28	35.9	8	27.6	15	36.6	
With spouse and children	66	44.6	32	41	11	37.9	23	56.1	
With friends	3	2	2	2.6	1	3.4	0	0	
**Has children**									0.31
Yes	118	78.7	62	79.5	20	69	36	83.7	
No	32	21.3	16	20.5	9	31	7	16.3	
**Self-reported health status**									
SF-12 physical score	144	69.5 (10.6)	74	69.8 (9.7)	29	71.7 (9.7)	41	67.3 (12.5)	0.17
SF-12 mental score	144	59.5 (14.8)	74	59.0 (14.5)	29	57.1 (16.4)	41	62.3 (14.0)	0.32
**HADS**									
Anxiety subscale score	150	6.1 (3.5)	78	6.6 (3.6)	29	5.8 (3.6)	43	5.5 (3.0)	0.18
Depression subscale score	149	3.1 (2.9)	77	3.5 (2.9)	29	2.2 (2.7)	43	3.1 (2.8)	0.06

Abbreviation: *SD* standard deviation

The mean SF-12 scores were 69.5 (SD = 10.6) and 59.5 (SD = 14.8) for the PCS and MCS domains, respectively. The mean HADS score for the anxiety subscale was 6.1 (SD = 3.5), and the mean HADS score for the depression subscale was 3.1 (SD = 2.9). The results do not reveal significant differences according to the different types of care levels for any of these characteristics.

### Professional and practice characteristics at the prison workplace

Table 2 shows the professional and practice characteristics of the sample. Most of the respondents were nurses (53.3%), followed by junior or senior physicians (19.3%) and psychologists (14%). Most of the respondents had regular contact with departments of the neighboring hospital (68.2%). Nearly three-quarters (70%) reported that they had worked over 10 years in the healthcare system, and one-third of the overall sample (32%) had worked in the same workplace for over 10 years. Nineteen professionals (12.7%) were early-career professionals (in practice < 4 years). Nearly half of the participants (46%) never had irregular eating hours, whereas 10 and 6% of them often and always did, respectively. Most of the surveyed professionals (71.8%) reported that they never worked at night. Significant differences in occupational status, years in practice, years in practice in the current workplace, regular contact with departments of neighboring hospitals, night work and irregular eating hours were found among the surveyed participants according to the 3 care-level settings. The proportion of professionals having irregular eating hours and night work was higher for the full-time hospitalization care level than for the other levels. The proportion of early-career professionals working in a prison was higher for part-time hospitalization than for the other two levels of care. In terms of exposure to violence, 52.7 and 65.3% reported that they were sometimes exposed to verbal and physical violence, respectively. One-third of the sample (36.7%) reported often being exposed to verbal violence. Seventeen professionals (11.4%) had a history of complaint procedures for aggression in the current workplace. The proportion of professionals exposed to verbal or physical violence was higher at the first and third care levels than at the second level.

**Table 2:** Professional and practice characteristics in the workplace

	Full Sample		Care Level 1		Care Level 2		Care Level 3		
	N = 150		N = 78		N = 29		N = 43		
Characteristic	N	%/mean (SD)	N	%/mean (SD)	N	%/mean (SD)	N	%/mean (SD)	*p value*
**Occupational status**									0.004
Junior/senior doctor	29	19.3	15	19.2	7	24.1	7	16.3	
Nurse/nursing auxiliary	80	53.3	40	51.3	10	34.5	30	69.8	
Health nurse manager	6	4	3	3.8	2	6.9	1	2.3	
Occupational therapist/physical therapist	3	2	2	2.6	0	0	1	2.3	
Hospital service agent	2	1.3	0	0	0	0	2	4.7	
Psychologist	21	14	15	19.2	6	20.7	0	0	
Medical secretary	9	6	3	3.8	4	13.8	2	4.7	
**Years in practice in the job**									0.008
< 4 years	19	12.7	5	6.4	10	34.5	4	9.3	
≥ 4 years and < 10 years	26	17.3	16	20.5	4	13.8	6	14	
≥ 10 years	105	70	57	73.1	15	51.7	33	76.7	
**Years in practice in the current workplace**									0.01
< 4 years	56	37.3	26	33.3	17	58.6	13	30.2	
≥ 4 years and < 10 years	46	30.7	25	32.1	2	6.9	19	44.2	
≥ 10 years	48	32	27	34.6	10	34.5	11	25.6	
**Hours worked per week**	148	37.2 (8)	77	36.5 (8.2)	29	38.1 (6.2)	42	37.9 (8.8)	0.57
**Irregular eating hours**									0.002
Never	69	46	40	51.3	20	69.0	9	20.9	
Seldom	57	38	29	37.2	6	20.7	22	51.2	
Often	15	10	5	6.4	2	6.9	8	18.6	
Always	9	6	4	5.1	1	3.4	4	9.3	
**Night work**									< 0.0001
Never	107	71.8	70	90.9	23	79.3	14	32.6	
Seldom	13	8.7	3	3.9	4	13.8	6	14	
Often	27	18.1	3	3.9	2	6.9	22	51.2	
Always	2	1.3	1	1.3	0	0	1	2.3	
**Exposure to verbal violence**									< 0.0001
Never	10	6.7	2	2.6	7	24.1	1	2.3	
Seldom	79	52.7	49	62.8	16	55.2	14	32.6	
Often	55	36.7	23	29.5	6	20.7	26	60.5	
Always	6	4	4	5.1	0	0	2	4.7	
**Exposure to physical violence**									0.09
Never	22	14.7	13	16.7	7	24.1	2	4.7	
Seldom	98	65.3	52	66.7	18	62.1	28	65.1	
Often	29	19.3	12	15.4	4	13.8	13	30.2	
Always	1	0.7	1	1.3	0	0	0	0	
**History of complaint procedures for aggression in the workplace** (yes)	17	11.4	11	14.3	3	10.3	3	7	0.52
**Training stage in prison during initial formation** (yes)	48	32.2	23	29.9	10	34.5	15	34.9	0.82
**Regular contact with departments of the local hospital** (yes)	101	68.2	55	70.5	24	85.7	22	52.4	0.01

Abbreviation: *SD* standard deviation

### Job conditions, job satisfaction and MBI profiles

The results of the Karasek, Effort-Reward Imbalance and MBI questionnaires are depicted in Table 3. Higher mean scores for psychological and physical job demands and lower mean scores for decision latitude (control) and social support indicate high levels of job stress. Job demands, job control and social support differed between care levels, with a higher mean score for job demands at the part-time hospitalization care level and lower mean scores for social support and job control at the full-time hospitalization care level. Twenty-six participants (18.6%) reported job strain, with no significant difference between levels of care. Three-quarters of the participants (75.3%) reported satisfactory links with prison services, and two-thirds (61.8%) were satisfied with the links with the integration and probation service. The proportion of workers who would continue working in the setting was lower at the part-time hospitalization care level than at the other levels (*p = 0.004).*

**Table 3:** Job conditions and satisfaction in the workplace

	Full Sample		Care Level 1		Care Level 2		Care Level 3		
	N = 150		N = 78		N = 29		N = 43		
Characteristic	N	%/mean (SD)	N	%/mean (SD)	N	%/mean (SD)	N	%/mean (SD)	*p value*
***Karasek scores***									
**Job demands**	144	21.3 (4.2)	73	21.8 (4.2)	29	22.2 (4.5)	42	19.8 (3.7)	0.02
**Job control**	144	72.8 (10.6)	76	75.5 (9.7)	29	74.9 (8.4)	39	65.9 (10.8)	< 0.0001
**Social support**	138	24.8 (4)	70	25 (3.7)	28	26.2 (4.4)	40	23.6 (4.2)	0.03
Job strain^a^	26	18.6	11	15.3	5	17.2	10	25.6	0.40
Isostrain^b^	12	9.0	5	7.5	3	10.7	4	10.5	0.78
***Effort-reward imbalance***									
Effort-Reward Imbalance ratio	143	0.4 (0.2)	75	0.5 (0.2)	28	0.5 (0.1)	40	0.4 (0.2)	0.09
Overcommitment	150	12.9 (3.8)	78	13.3 (4)	29	12.9 (3.7)	43	12.2 (3.6)	0.43
***Burnout scores***^a^									
**Emotional exhaustion**	145	13.1 (10)	77	14 (10.6)	28	12.7 (10)	40	11.5 (8.7)	0.55
**Depersonalization**	148	6.7 (5.4)	78	7.6 (5.7)	29	5.3 (5.9)	41	5.9 (4.2)	0.07
**Personal accomplishment**	142	34.7 (8)	74	36.4 (7.1)	28	35.5 (6.6)	40	31.3 (9.5)	0.04

Abbreviation: *SD* standard deviation

^a^ Job strain: work situation when the psychological demand is greater than the median and the decision latitude is less than the median

^b^ Isostrain: work situation combining a job strain situation with social support below the median level

**Fig. 1:**
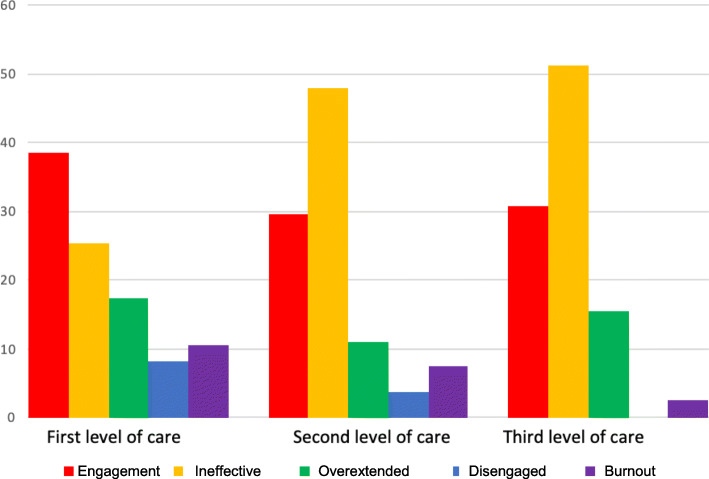
Distribution of MBI profiles among professionals according to the three levels of care

According to the Maslach criteria, which consider burnout syndrome to be present when all three dimensions are severely abnormal, the prevalence of the burnout profile was 7.8%. One-third of the sample (36.9%) presented an ineffective profile; 15.6 and 5% had overextended and disengaged profiles, respectively. Although the proportion of ineffective MBI profiles was higher at the part-time and full-time hospitalization levels and the proportion of disengaged and burnout profiles was higher at the first care level, no significant difference in the overall MBI profiles was observed based on the practice setting. The repartitioning of the five profiles among the three levels of care is depicted in Fig. 1. The PA scores were significantly different between the levels of care (*p = 0.04*).

Based on the results presented in Table 4, some features could be described for each MBI profile. Significant differences in the five MBI profiles are shown in regard to occupational status, history of complaint procedures in the current workplace, irregular eating hours, anxiety and depression and the mental health component in regard to quality of life. Other tendencies based on Table 4 are described in Table 5. Thus, in regard to the burnout profile, professionals were younger, were more often childless and more often had been in the workplace for less than 10 years. Physicians who had irregular eating hours due to their work and those with a history of complaint procedures for aggression in the workplace were more concerned about situations with job strain and were more frequently anxious and depressive. These health professionals presented lower mean scores on the SF-12 mental component. Most professionals reported unsatisfactory links with prison services.

**Table 4:** Prevalence and characteristics of the five MBI profiles of the sample

	Full Sample		Engagement		Ineffective		Overextended		Disengaged		Burnout		
	*N* = 141		*N* = 49		*N* = 52		*N* = 22		N = 7		*N* = 11		
			(34.8%)		(36.9%)		(15.6%)		(5.0%)		(7.8%)		
	N	%/mean (SD)	N	%/mean (SD)	N	%/mean (SD)	N	%/mean (SD)	N	%/mean (SD)	N	%/mean (SD)	*P value*
**Sex**													NS
Male	37	26.4	13	26.5	10	19.6	6	27.3	3	42.9	5	45.5	
Female	103	73.6	36	73.5	41	80.4	16	72.7	4	57.1	6	54.5	
**Age**	139	42.8 (11.2)	49	43.1 (11.7)	50	43.1 (11.3)	22	44.2 (10.8)	7	43.4 (9.4)	11	37.5 (9.7)	NS
**Marital status**													
Never married	19	13.5	4	8.2	10	19.2	4	18.2	0	0.0	1	9.1	
Married/live with a partner	108	76.6	41	83.7	37	71.2	14	63.6	7	100.0	9	81.8	
Separated/divorced/widowed	14	9.9	4	8.2	5	9.6	4	18.2	0	0.0	1	9.1	
**Occupational status**													0.01
Junior/senior doctor	27	19.1	12	24.5	5	9.6	4	18.2	2	28.6	4	36.4	
Nurse/nursing auxiliary	77	54.6	26	53.1	34	65.4	9	40.9	3	42.9	5	45.5	
Occupational therapist/physical therapist	3	2.1	2	4.1	0	0.0	1	4.5	0	0.0	0	0.0	
Medical secretaries	8	5.7	0	0.0	8	15.4	0	0.0	0	0.0	0	0.0	
Health nurse manager	6	4.3	2	4.1	2	3.8	1	4.5	1	14.3	0	0.0	
Psychologist	20	14.2	7	14.3	3	5.8	7	31.8	1	14.3	2	18.2	
**Years in practice in the job**													NS
< 4 years	17	12.1	5	10.2	7	13.5	1	4.5	1	14.3	3	27.3	
> = 4 years and < 10 years	26	18.4	9	18.4	7	13.5	5	22.7	1	14.3	4	36.4	
> = 10 years	98	69.5	35	71.4	38	73.1	16	72.7	5	71.4	4	36.4	
**Exposure to physical violence**													NS
Never	19	13.5	6	12.2	8	15.4	3	13.6	1	14.3	1	9.1	
Seldom/often/always	122	86.5	43	87.8	44	84.6	19	86.4	6	85.7	10	90.9	
**Exposure to verbal violence**													NS
Never	10	7.1	2	4.1	7	13.5	1	4.5	0	0.0	0	0.0	
Seldom/often/always	131	92.9	47	95.9	45	86.5	21	95.5	7	100.0	11	100.0	
**History of complaint procedure for aggression in the workplace**													0.02
No	123	87.9	43	87.8	47	92.2	21	95.5	6	85.7	6	54.5	
Yes	17	12.1	6	12.2	4	7.8	1	4.5	1	14.3	5	45.5	
**Irregular eating hours**													0.05
Never	66	46.8	29	59.2	26	50.0	6	27.3	4	57.1	1	9.1	
Seldom	51	36.2	16	32.7	18	34.6	9	40.9	2	28.6	6	54.5	
Often	15	10.6	3	6.1	4	7.7	4	18.2	1	14.3	3	27.3	
Always	9	6.4	1	2.0	4	7.7	3	13.6	0	0.0	1	9.1	
**Link with prison staff**													NS
Highly satisfactory/satisfactory	105	75.5	37	75.5	43	84.3	14	66.7	5	71.4	6	54.5	
Little or not at all satisfactory	34	24.5	12	24.5	8	15.7	7	33.3	2	28.6	5	45.5	
**HADS-Anxiety subscale**													< 0.0001
No	123	87.2	49	100.0	47	90.4	16	72.7	7	100.0	4	36.4	
Yes (score > = 11)	18	12.8	0	0.0	5	9.6	6	27.3	0	0.0	7	63.6	
**HADS-Depression subscale**													0.03
No	137	97.9	49	100.0	51	100.0	20	90.9	7	100.0	10	90.9	
Yes (score > = 11)	3	2.1	0	0.0	0	0.0	2	9.1	0	0.0	1	9.1	
**Karasek**													
Job Strain	24	17.9	5	11.1	8	16.0	4	19.0	1	14.3	6	54.5	NS
Isostrain	11	8.6	1	2.4	4	8.3	3	15.0	1	14.3	2	18.2	NS
**Effort-reward imbalance**													
Effort-reward imbalance ratio > 1	2	1.5	0	0.0	0	0.0	2	9.1	0	0.0	0	0.0	0.06
**Self-reported health status**													
SF-12 physical score	136	69.8 (10.2)	47	71.9 (8.2)	50	70.5 (10.0)	22	65.6 (11.4)	7	68.3 (13.8)	10	66.2 (12.1)	NS
SF-12 mental score	136	59.3 (15.1)	47	67.6 (7.4)	50	62.1 (12.4)	22	42.2 (13.8)	7	65.6 (12.0)	10	38.8 (12.5)	< 0.0001

Abbreviations: *SD* standard deviation; *NS* non significant

**Table 5:** Overview of the specific characteristics and tendencies of the MBI profiles

	Engagement	Ineffective	Overextended	Disengaged	Burnout
**Sex**	female	female	female	50/50	50/50
**Age**	> 40	> 40	> 40	> 40	< 40
**Marital status** NS^a^	/	/	/	/	/
**Occupational status**	Nurse	Nurse	Psychologist	Health manager and doctor	Doctor
**Years in practice in the job**	> = 10	> = 10	> = 10	> = 10	< 10
**Exposure to physical violence** NS^a^	/	/	/	/	/
**Exposure to verbal violence** NS^a^	/	/	/	/	/
**History of complaint procedure for aggression in the workplace**	No	No	No	No	50/50
**Irregular eating hours**	Never/seldom	Never/seldom	Never/seldom	Never/seldom	Seldom/often
**Link with prison staff (satisfactory)**	yes	yes	yes	yes	50/50
**HADS-Anxiety subscale**	No	No	No	No	Yes
**HADS-Depression subscale**	No	No	Yes	No	Yes
**Karasek job strain**	Low	Low	Low	Low	High
**Karasek isostrain** NS^a^	/	/	/	/	/
**Effort-reward imbalance > 1** NS^a^	No	No	Yes	No	No
**Self-reported health status PS** NS^a^	/	/	/	/	/
**Self-reported health status MS**	> 60	> 60	< 50	> 60	< 40

NS^a^ nonspecific

## Discussion

This study is among only a few to investigate burnout in professionals working in units providing health services to inmates [42] and the first to characterize these workers according to MBI profiles. First, our results indicate that the most frequent profiles are ineffective and engagement, which constituted 71% of the sample. These findings are consistent with the profiles identified in a previous study among healthcare employees [15] but with a few differences, as the most prevalent MBI profile was ineffective rather than engagement in our sample. This ineffective profile reflects a psychological relationship with work in which a person is not distressed but also not fully engaged, lacking the fulfilling qualities of engagement that are defined by “energy, involvement and efficacy” [43]. The experience of being ineffective does not coincide with high rates of exhaustion or high levels of cynicism. Instead, it reflects a loss of confidence in one’s capabilities, perhaps as a result of an environment that offers little recognition for a job well done or for work that feels tedious or. This experience is far more common among nurses or nursing auxiliaries in our sample. The ineffective profile clearly appears more negative than engagement but preferable to the distress inherent in the burnout, overextended and disengaged conditions [15].

Our finding indicates a 7.8% prevalence of burnout, which is in line with previous studies when all three dimensions (EE, DP and PA) are severely abnormal in penitentiary settings [44] and consistent with the fact that the engaged profile is four times less common than the engaged profile among healthcare employees [43]. However, this result is well below the high burnout prevalence rates that have been previously reported among French health professionals, ranging from 28 to 73% [45–47]. Methodological differences could influence these reported burnout rates. There is real controversy in the literature regarding the tools to measure burnout and which dimensions of the MBI to include, with studies using one [48–50], two [51, 52] or all three [7, 29] dimensions to classify burnout. With the ineffective profile, the overextended and disengaged profiles reflect transitional states toward burnout and are thus cause for concern. Five percent of our participants met the classification for the disengaged profile, with high cynicism; this figure is below the proportion previously identified for this profile among healthcare providers [15].

In line with a previous study using latent profiles [53], the MBI profiles did not differ regarding sex even if participants who identified as male were more likely to be classified as having burnout and disengaged profiles than those who identified as female. Professionals with a history of complaint procedures for aggression and with regular experience of verbal aggression were likely to experience a high level of cynicism. Professionals in units treating inmates are particularly exposed to intimidation, aggression and rebellion, which are known to lead to psychosocial risks [54]. One source of cynicism and therefore disengagement could be the transition from an idealistic world of a healthcare provider to the real world of threats and exposure to physical and verbal violence despite providing care. The 15.6% prevalence of the overextended profile is in line with the findings of Leiter and Maslach [15], and the prevalence of psychologists experiencing high levels of exhaustion in correctional settings is in line with the findings in previous work [55]. This result supports the need for workload adjustments for professionals who are involved but exhausted.

Knowledge of these profiles can be useful when designing interventions focused on both people and job situations, as the development of burnout syndrome is influenced by structural work environment factors such as job demands, low ability to exert control and unsupportive workplaces [56]. At the organizational level, offering a sustainable workload and increasing rewards by providing more choices may be suggested for overextended individuals. An ineffective individual may benefit from more recognition and rewards, and a disengaged individual may require a supportive work community and/or clear value and meaningful work. At the individual level, previous studies among workers experiencing challenging situations have emphasized increasing resilience, which can be developed [52, 57]. Resilience is considered to be the ability to adapt successfully in the face of trauma, adversity, stress, significant threat or tragedy [58]. Resilience can help professionals sustain the capacity to not be disrupted by threats or stress and to stay engaged at work, as previously described [59]. As work-related stress is a real public health concern and might play a role in the development of mental health problems in healthcare professionals [28], the high prevalence of anxiety and depression symptoms among individuals with the overextended and burnout profiles supports the conclusion that a number of steps should be taken at the individual level to promote wellness. Early detection and prevention are needed to help counteract the stressors inherent in the workplace and the associated negative impacts on mental health to maintain a high level of mental well-being in this demanding workplace.

Although the MBI profiles did not differ significantly based on the type of care level, our research showed that professionals from the first level of care were more susceptible to burnout, while those from the second and third levels of care were more likely to fit the ineffective profile. For the second and third care levels, the findings emphasize the important roles of esteem, recognition and appropriate feedback in building engagement. Improvements in work environments in the first level of care, including having respectful working relationships with other service providers, being attentive to colleagues and anticipating the impact of one’s behavior on others, as well as clear targets, strategic leverage points and regular organizational assessments, could help to prevent burnout.

Several methodological limitations should be discussed. First, a small number of professionals were included in this study, which prevented us from using statistical tools such as a multivariate polytomous logistic regression model. This could be a next step for future research. Second, our findings may not be fully representative of professionals working in units for inmates and may not be generalizable to other groups, as professionals voluntarily decided to participate. Third, the data were collected using self-report questionnaires, which, although anonymous, may introduce bias in the direction of socially desirable responses. However, to our knowledge, this study is the first to assess the profiles of French professionals, and they are expected to follow the recommendations. This method for the classification of participants according to MBI profiles is relatively recent in the long history of the MBI, and this approach could be helpful for the earlier recognition of individuals who may be at risk of developing burnout. Moreover, this is the first study to pay attention to professionals working among the three different levels of care in a detention setting (ambulatory and part-time and full-time hospitalization). Furthermore, our results indicate that initiatives for professionals should include improved guidance for younger workers in units for inmates. Developing resources to facilitate exchanges with partnerships and to build a better work environment is essential, as these actions could afford mental health benefits.

## Conclusions

Our findings suggest that both organizational and individual factors need to be addressed to reduce the high prevalence of disengaged, overextended and ineffective intermediate profiles on the engagement-burnout continuum. The findings emphasize the importance of a more customized approach to interventions, and future solutions may need to take into account the key underlying problems for different groups of people. Our findings also support the need to reflect on and discuss the context of public policies to help these professionals, who deserve to be better assisted. As burnout research in the correctional setting grows, future research in the form of longitudinal studies would be useful in investigating how profiles develop and change over time and how interventions can be used to impede the development of burnout and mitigate its potential negative consequences.

## Data Availability

A de-identified dataset may be made available upon reasonable request of the corresponding author.

## Abbreviations

EHCAU: Evaluation of Health CAre in Units for inmates
HRQoL: Health-related quality of life
MBI: Maslach Burnout Inventory
SF-12: Short Form-12 questionnaire
HADS: Hospital Anxiety and Depression Scale
